# A novel three-dimensional smile analysis based on dynamic evaluation of facial curve contour

**DOI:** 10.1038/srep22103

**Published:** 2016-02-25

**Authors:** Yi Lin, Han Lin, Qiuping Lin, Jinxin Zhang, Ping Zhu, Yao Lu, Zhi Zhao, Jiahong Lv, Mln Kyeong Lee, Yue Xu

**Affiliations:** 1 Department of Oral and Maxillofacial Surgery, Guanghua School of Stomatology, Hospital of Stomatology, Sun Yat-sen University, Guangdong Provincial Key Laboratory of Stomatology, Guangzhou 510055, P. R. China; 2 Department of Orthodontic, Guanghua School of Stomatology, Hospital of Stomatology, Sun Yat-sen University, Guangdong Provincial Key Laboratory of Stomatology, Guangzhou 510055, P. R. China; 3 Department of Medical Statistics and Epidemiology, School of Public Health, Sun Yat-sen University, Guangzhou 510055, P. R. China; 4 School of Data and Computer Science, Guangdong Provincial Key Laboratory of Computational Science, Sun Yat-sen University, Guangzhou 510275, P. R. China; 5 Division of Dentistry and Orthodontics, Children’s Hospital Los Angeles, 4650 Sunset Blvd, #116 Los Angeles, CA 90027.

## Abstract

The influence of three-dimensional facial contour and dynamic evaluation decoding on factors of smile esthetics is essential for facial beauty improvement. However, the kinematic features of the facial smile contour and the contribution from the soft tissue and underlying skeleton are uncharted. Here, the cheekbone-maxilla contour and nasolabial fold were combined into a “smile contour” delineating the overall facial topography emerges prominently in smiling. We screened out the stable and unstable points on the smile contour using facial motion capture and curve fitting, before analyzing the correlation between soft tissue coordinates and hard tissue counterparts of the screened points. Our finding suggests that the mouth corner region was the most mobile area characterizing smile expression, while the other areas remained relatively stable. Therefore, the perioral area should be evaluated dynamically while the static assessment outcome of other parts of the smile contour contribute partially to their dynamic esthetics. Moreover, different from the end piece, morphologies of the zygomatic area and the superior part of the nasolabial crease were determined largely by the skeleton in rest, implying the latter can be altered by orthopedic or orthodontic correction and the former better improved by cosmetic procedures to improve the beauty of smile.

Smile esthetics are determined by a harmonious relationship among different components, such as facial skeleton, musculature, fat distribution, and skin texture^1^. In its natural condition, the face appears as a stereoscopic dynamic structure with a smooth contour and shadowing between different anatomical regions. The three promontories, namely the nose, malar zygomatic eminences, and chin-jaw-line^2^, along with the muscle attachments, delineate the facial topography. In the various degrees of smile, soft tissues undergo deformation and translocation following muscle contractions that are controlled by an intricate neural mechanism. In a static condition, facial appearance even changes in its three-dimensional (3D) configuration, symmetry, and proportion under the shift of ambient light during observation^3^.

With increasing esthetic demands from the public, an objective evaluation of three-dimensional facial contour and smile analysis is necessary^4^,^5^,^6^. Various studies^7^,^8^,^9^ have been conducted to recognize zones or landmarks of the soft tissue to represent distinct anatomical characteristics in movement. From the frontal view, a smiling face is marked by a prominent curve connecting the inner outline of the malar fat pad and the corner of the mouth transversely and inferiorly. Arnett *et al.*^10^,^11^ first described a smooth curve called the “cheekbone contour” that begins anterior to the ear, extends through the cheekbone point (CP) anterior-inferiorly, and ends at the maxilla point (MxP) adjacent to the alar base of the nose. This curve can be observed both in frontal and profile views. Arnett and Bergman have stated its importance in evaluating the anteroposterior relationship of the maxilla and mandible. From the MxP, another prominent line in the face, called the nasolabial fold, begins and curves downward and bilaterally to be lost finally below and lateral to the oral commissure^12^. The nasolabial fold is composed of deep, firm fibrous tissue that receives terminal muscle fibers from the levator labii superioris muscle and serves as the fibrous origin for the “fold muscles”^13^,^14^. At the formation stage of smiling, the levator muscles raise the lip and nasolabial fold upward by conquering the resistance of cheek fat. Whenever the muscles contract, the lip is pulled up and the fold deepens^13^.

Natural continuation of the cheekbone contour and nasolabial fold depicts the most prominent contour in a human face from different angles. We termed the combination of these two facial lines as “cheekbone-nasolabial contour” or “smile contour” and examined the relationship between this contour in space and the smile esthetics. This smile contour emerges more prominently when smiling, delineating the overall facial topography of different individuals.

Three-dimensional (3D) facial motion captures have long been used in facial expression animation and identification. Recently, most researchers have used 3D motion analyzers for noninvasive quantification of facial movements^15^,^16^,^17^,^18^. Some of the studies have addressed the use of the 3D facial motion capture in the diagnosis and treatment of cleft lip repair^19^. To date, 3D facial motion capture analyses are based on individual landmarks. A major limitation of using landmarks in 3D analysis is potential interference between adjacent landmarks located too close to each other during the capture process. However, facial esthetics can be better featured by continuous 3D curve contour rather than discrete landmarks. Thus, the evaluation of continuous lines or curves is more applicable and clinically meaningful.

In our preliminary studies, stable points on the facial smile contour were identified by kinematic analysis during facial expressions. The aims of this study were to propose a promising indicator in clinic smile analysis, to determine kinematic features of the facial smile contour and to relate the underlying skeletal and soft tissue contributions to the facial smile contour in Chinese youth. A method combining the expression capture and mathematic model calculation was therefore proposed, and the functional formula of the smile contour in static, smiling and laughing conditions were set forth. We hypothesized that the movement amplitude varied for different parts along the smile contour, and the soft tissue configuration attributed differently to various facial components – mainly the soft tissue and the underlying hard tissue – diversifying the smile beauty of individuals. Determining the patterns and range of movement along the cheekbone-nasolabial curve in normal subjects will elucidate individual variations in smile esthetics. In addition, any association between the skeletal and soft tissue will help identify anatomic regions that may be altered using surgical or medical interventions to improve the morphology and dynamics of smile. This study will provide clues and hints for the treatment planning in clinic of orthodontics, orthopedic or plastic surgery when screening the determination areas and quantifying the modification volumes.

## Methods and Materials

### Participants

A total of 80 students at Sun Yat-sen University with no previous orthodontic treatments were enrolled for the study of Chinese youth. The subjects were closely matched with respect to gender and age. This study was approved by the Ethics Committee of the Hospital of Stomatology, Sun Yat-sen University and adhered to the tenets of the Declaration of Helsinki. Written informed consents for the adhesion of retro-reflective markers, collection of expression movement, storage of the photograph and radiograph and publication of this study were obtained before data collection after a complete verbal explanation.

Inclusion criteria for the subjects were: skeletal Class I (straight profile), 18 to 28 years of age, and normal mandibular function. Exclusion criteria were: facial muscle functional disorders, facial injury and scar, facial paralysis, deformities, hypersensitivity, and other defects.

### Curve contour drawing and landmarks location

The experiments took place in a quiet room with normal indoor lighting. Participants were comfortably seated in an armchair and were asked to relax and keep their eyes open. Head movement contaminations of the kinematic recordings of the facial movements were avoided using a head holder. A video-based tracking system (Natural Point Arena Expression, Inc., USA) was used to track the movement of retro-reflective markers measuring 3 mm in diameter and secured to pre-determined facial landmarks using hypoallergenic adhesive tape. Three soft tissue landmarks – which are definable and used to define the terminals and turning point of the curve – were positioned firstly to assist in curve drawing (Table 1). The locating method for the cheekbone point (CP), the maxilla point (MxP), and the mandible point (MdP) can also be found in Table 1. Given the size of the landmarks and the identification accuracy of the system, the landmarks were located evenly at 8 mm intervals. Using the landmarks, the cheekbone-maxilla-nasolabial curve, consisting of the cheekbone contour and nasolabial fold, was drawn bilaterally on each subject.

### Three-dimensional dynamic data collection

The volume in which each subject’s head was captured was calibrated using an “L”-shaped frame. The L frame had three markers fixed to known locations on the frame. Next, a research assistant waved a wand with precisely located markers throughout the capture volume. This process located the exact camera positions, accurately measured the focal lengths, and accounted for any geometric distortions in the camera lenses. The error in specifying marker positions within the system was measured to be 0.3 mm. Finally, each participant was positioned within the calibrated area and the cameras adjusted to focus on the face. To facilitate accuracy and the reproducibility of this position, each participant was asked to sit still on a chair with his or her back supported.

In this research, the smile expression refers to the posed smile. The smile expression was studied while participants were watching a sequence of pictures displayed on a PC screen placed at the eye level, 70 cm in front of them. Pictures represented the face of 2 actors and 3 actresses (age range: 20–30 years) performing a Duchenne posed smile. A randomized sequence of 10 pictures was presented to the participants (pictures representing the face of each actor were presented twice). Pictures were shown for 5s each and were interposed by a black screen for an equally long period of time. Posed smiling was studied by asking the subjects to mimic the expression of the faces presented on the PC screen. The laughing expression refers to the extreme smile with the mouth stretching wide laterally to the maximal extent. After being asked to mimic the posed smile, the subjects were required to extend their smile to the maximum extent previously stated as extreme smile. The subjects were asked to laugh three times and maintain each for 3 seconds at a 3-second static interval. The three-dimensional movement data of landmarks was captured at a rate of 100 frames per second. Simultaneously, a synchronized video camera recorded the facial movements. Two research assistants reviewed the tracked data to identify and isolate groups of movements or “epochs” for each subject during smiling and laughing. Generalized Procrustes analysis was used to remove whole-head motion and scale out head size differences among the participants.

### Evaluation of movement characteristics and specific landmarks screening

For each participant, 40 seconds of video data was recorded. The video tracking software program calculated the X, Y, and Z coordinates of the centers of every landmark, and the distance of one point at two epochs representing the static and smile, or laugh, condition respectively. With these data, all the coordinates were normalized to [−1, 1], and the polynomial function was used to conduct the curve-fitting program. After comparing the performance of fitting and parsimony among quadratic, cubic, quartic, and even quintic polynomial functions, the cubic function stood out from the competition. Then the chosen function model was set as follows:


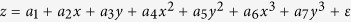


where z = the value of the Z axis, a1–a7 = parameters, x = the value of the X axis, and y = the value of the Y axis.

For the estimation of coefficients in the above function, we used the genetic algorithm (GA) to optimize the estimated process to obtain the global optimum solutions^20^ since the frequently used method of ordinary least squares may only lead to a locally optimum solution. The sum of squared error was selected as the fitness function in GA.

Mean maximum amplitude (in cm) was calculated to characterize the dynamic features and stability of the landmarks using custom-made scripts in Matlab R2014a (The MathWorks, Inc, USA). Mean maximum amplitude is the average distance of a landmark at two epochs between facial expression and static state. The length-amplitude curves were drawn and underwent a Kernel Smoother^21^ to obtain continuous and smooth curves. Therefore, specific landmarks during expression movement on the curve were screened out.

### Three-dimensional reconstruction and analysis

The samples for three-dimensional analysis consisted of the cone beam computed tomography (CBCT) scans of all the subjects in habitual occlusion with the lips closed. The DCT Pro CBCT device (Vatech, Co., Ltd., Hwasung, Korea) was set at a 20 × 19 cm field of view, 24-second scanning time, and 0.4 voxel scanning resolution. The image consisted of an area from the highest point on the superior margin of the cranial vault, to the inferior border of the mandibular body. The gross data and the obtained slices were imported into and reconstructed in a three-dimensional model by an interactive image analysis system (Mimics, 14.0, Materialise, Leuven, Belgium). The origin of the xyz coordinate system was determined automatically once the data were imported.

The specific landmarks were relocated on the facial surfaces of every subject before three-dimensional photos (3dMD, Atlanta, GA) were taken for transformation of the location of these landmarks to the CBCT reconstructed soft tissue model. The three-dimensional coordinates of these soft tissue points and the facial hard tissue points were imported into the Matlab Compiler for the position of corresponding hard tissue points by the homeomorphic mapping principle^22^. Let *A* denote the selected hard tissue feature point set, and *B* the soft tissue point set. A mapping from *A* to *B* was defined as


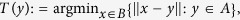


where ‖.‖ is the Euclidean norm in the three-dimensional Euclidean space. In this way, specific points on the facial soft tissue and their hard tissue counterparts were obtained for further analysis.

### Statistical Analysis

Canonical correlation analysis was used to calculate the correlation between soft tissue points and corresponding hard tissue points. Squared canonical correlations represent the contribution of hard tissue positions to the soft tissue counterparts. All the statistical analyses were conducted using SPSS 17.0 software.

## Result

**Figure 1:**
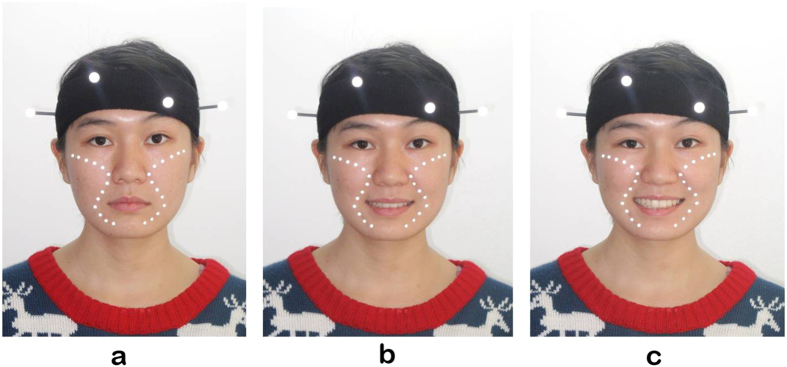
Landmarks location. Affixing 20–26 reflective markers on the facial surface along with the cheekbone-maxilla-nasolabial curve at 0.8 cm interval evenly. The number of landmarks varied with the facial size and the length of the curve. The three-dimensional coordinates of markers were translated in a local coordinate system created by four head landmarks and subsequently exported. The landmark locations under (**a**) static, (**b**) smile and (**c**) laugh condition were hereby shown.

Of the 80 subjects, five were excluded according to the criteria – two were diagnosed as skeletal type III and mandibular deviation, two had received orthodontic treatment, and one was allergic to the adhesive agent. Since subjects varied in their face sizes, 10 to 13 landmarks were limited to each side of the contour line (Fig. 1). The curve-fitting procedures were conducted in static, smile, and laughing positions, and three curve-fitted equations were constructed. The coefficient estimates are shown in Table 2.

**Figure 2:**
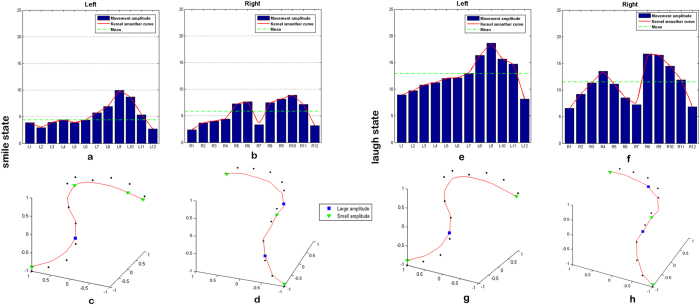
The motion amplitude of Cheekbone-Maxilla-Nasolabial contour. **(a,b**) Length-amplitude curve of the contour after Kernel Smoother in left and right side at smiling, (**c,d**) Curve-fitting results and the relocation of typical points on the left and right contour at smiling, (**e,f**) Length-amplitude curve of the contour after Kernel Smoother in left and right side at laugh condition, and (**g,h**) Curve-fitting results and the relocation of typical points on the left and right contour at laugh condition.

To display the displacement of different locations along the smile contour, we selected a standard case (with natural smile contour in medium esthetical level judged by two clinicians) as a reference. The length-amplitude curves of the facial contour of this standard case, with 12 points on each side, were depicted after Kernel smoothing. Subsequently, the specific points with large or small movement amplitude were identified and positioned on the fitted curve. During smile (Fig. 2a–d), the length-amplitude curves of both sides have double peaks, especially on the right side. The location of the first peak is inconspicuous on the left side but apparent on the right side, between R5 and R6. The second peak is prominent bilaterally between L9/R9 and L10/R10, which corresponds to the corner of the mouth. For stable points, both the origin and terminus of the contour curve displayed small amplitude during smiling. Specifically, we also noticed a valley amplitude point in the middle of the right curve (R7). As for the laughing position (Fig. 2e–h), the differences between the length-amplitude curves of the two sides were more evident, shown as a one-peak form on the left side and two-peak forms on the right. However, the changing rule along the bilateral fitted curve is similar to the smile condition. The position of points with large and small movement amplitude resemble each other, except for mildly shifting forward. Both peak between L8/R8 and L9/R9, followed by the second peak at R4 on the right.

**Figure 3:**
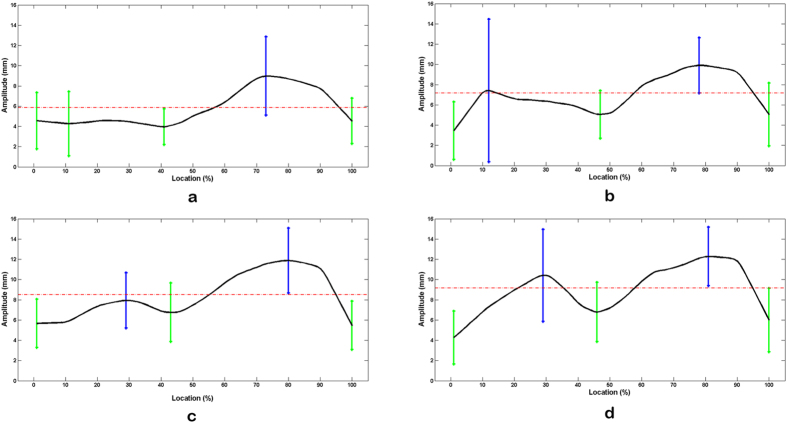
The average length-amplitude curve of Cheekbone-Maxilla-Nasolabial contour. The length from origins to specific landmarks was drawn on the horizontal axis whereas the motion amplitude was drawn on the vertical axis. The vertical bars represent the location of specific landmarks and the length of them represent the standard deviation of the motion amplitude at each landmarks. Green bars are located at the small amplitude points and blue ones are located at the large amplitude points. (**a**) Left side in smile state, (**b**) right side in smile state, (**c**) left side in laugh state and (**d**) right side in laugh state.

To confirm the relative location of specific points with the largest or smallest movement amplitude in smiling, the total length of the smile contour was normalized to 100% and the locations were denoted as the relative distance away from the original points of the contour. Length-amplitude curves were constructed after Kernel smoothing. The precise position of these points is shown in Table 3 with error bars in Fig. 3. Similar to the standard case, the shapes of the length-amplitude curves were not symmetric. The movement amplitude at specific points and the mean amplitude are both larger on the right compared to the left side. The standard deviation at the first peak of the right curve, however, is too large to be dependable. Otherwise, the tendency of the right curve is basically the same as the left. In the laughing state, the average curves had two peaks bilaterally, with general movement amplitude and the range of movement both larger on the right than those on the left. The positions of the specific points were similar to those of the smile state except for L2′/R2′. The first amplitude peak shifted downward from the smile to the laughing state.

**Figure 4:**
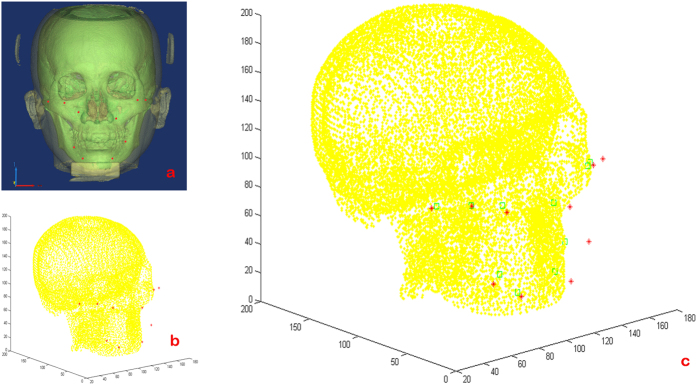
Orientation of the hard tissue landmarks corresponding to the specific points on the soft tissue. (**a**) The three-dimensional reconstructed model of craniofacial tissue and the located specific points over the soft tissue, (**b**) importing the hard tissue point cloud and the coordinates of specific point data into MATLAB software, and (**c**) the corresponding hard tissue points were obtained by homeomorphic mapping using this software.

Specific points relocated to the CBCT-reconstructed soft tissue model are also shown in Table 3. The pinpoint of these specific points came from their average curve lengths from the origin in smiling and laughing. The corresponding points on the surface of the hard tissue were subsequently obtained by homeomorphic mapping method using Matlab Compiler (Fig. 4). The points L1′, L2′, R1′, and R2′ fell on the zygomatic arch area. L2′/R2′ and L3′/R3′ mapped to CP and MxP, respectively. R3′ was at the upper lateral of MxP. L4′/R4′ mapped to the corner of the mouth, with R4′ slightly downward. Finally, L5′/R5′ mapped to MdP.

On the basis of canonical correlation analysis (Table 4), there was a signification correlation between the soft tissue and hard tissue positions except at L5′/R5′. Of note, the hard tissue at L2′, R2′, and R3′ were strongly correlated to the soft tissue. The position of the soft tissue area around the mouth corner (L4′/R4′) displayed large amplitude in motion, but was closely related to the underlying hard tissue in the static state.

## Discussion

Smile analysis is not just functional evaluation, but esthetic assessment. Smile is a positive expression conducted by the stretching and contraction of mimetic muscles. Due to its social communicative value, facial smile evaluation has become a common concern of esthetic-related disciplines. The smile contour we selected to study is a natural continuation facial esthetic curve, depicting the most prominent contour in a human face, especially when smiling. The morphology and motional characteristics are crucial in static and dynamic evaluation of this contour, providing hints about the modified zones and volume for orthodontic or surgical treatment planning.

Dynamic analysis of the smile commonly refers to the Facial Action Coding System (FACS)^23^, which is anatomically based and describes minimal units of facial movement called Action Units (AUs) with descriptions of the resulting changes in facial appearance. Although FACS can detect the visual appearance changes during facial movement, including smiling and laughing in addition to identifying the relationship between musculature and observable movement, it is an assessment merely of expression movement. The information gathered by means of FACS is discrete and untargeted. However, the emphasis of our research stems from the clinical evaluation and diagnosis of smile esthetics, which means the smile contour should be analyzed specifically. Unfortunately, a large majority of this curve contour lies on the junction and boundaries of the AUs of FACS, which made it an inappropriate tool for this research. By directly collecting the real-time 3D movement data and 3D reconstruction, we can exclusively concentrate on the continuous structure from an overall perspective, without the restriction of separating units. Additionally, the result of these studies can be applied directly to determine the treatment planning.

On the other hand, traditional facial soft tissue analyses are based on the profile view, neglecting the frontal view, with which most people associate their self images^24^,^25^,^26^. Mackley^27^ has demonstrated that a profile is not a reliable predictor of the appearance of a person’s smile. Yet both the frontal and profile views cannot display all the esthetic predictors of a face, implying a 3D requirement for smile analysis. Since FACS study is based on two-dimensional picture sets, it can only be observed from a specific angle, which contributes to in inapplicability of FACS for this research. In this study, we analyzed the dynamic features of the points on the curve three-dimensionally. The curve-fitting equations of this contour in static, smile, and laughing states were constructed to specify the 3D and dynamic morphologies. The equations we obtained can be optimized by further dynamic studies on this contour curve and may contribute to the improvement in the analysis of facial esthetics in three dimensions.

The changing rules of the movement amplitude along the contour of two sides are not always symmetric in individual cases. On average, however, the fluctuation patterns are more synchronous bilaterally with the exception of L2′/R2′ in the smile state, where the right data are considered undependable. The mean amplitudes of the curve were larger during laughing than smiling, due to larger flexion of facial muscles in laughter. Greater amplitudes on the right side in both smile and laughing states are consistent with the valence hypothesis^28^, which holds the view that the left hemisphere of the brain controls positive emotions resulting in more intense expression of positive emotions – such as smiling and laughter – on the right side of the face. Therefore, extra-oral photographs should be collected bilaterally if dynamic smile analysis were being conducted to formulate a plan for esthetic treatment.

Facial soft tissue landmarks were more variable than their hard tissue counterparts. The greatest amount of soft tissue movement occurred at the oral commissure (L4′/R4′), followed by the middle part of the cheekbone contour (L2′/R2′). The most stable sections were found in the origins (L1′/R1′), around MxP (L3′/R3′) and MdP (L5′/R5′). The unstable points show a great relationship between the smile and laughing motions, while the stable points show the opposite. These areas are closely related to the smile or laugh and diversify the smile esthetics of individuals. The results shed light on the facial region whose underlying hard tissue position can be reliably judged from the clinical observation of the soft tissue contour. Therefore, when we are evaluating the dynamic characteristics with a static picture, it will be better to evaluate the unstable areas under the smile posture.

Although the soft tissue morphology is generally known to reflect the underlying hard tissue structure, the surface morphology of the hard tissue structure may not be fully expressed on the soft tissue images. In our preliminary studies, all the three-dimensional positions of specific soft tissue points (L1′/R1′–L5′/R5′) – with the largest or smallest movement amplitude on the bilateral smile contour – are mainly attributed to the position of hard tissue except for that of MdP. This indicates that the soft tissue configuration at this junction is variable as the hard tissue position changes. MdP is the junction of cheek, lower lip, and chin, and lies in the inferior margin of the orbicularis oris and depressor labii inferioris muscles, where the thickness of soft tissue is quiet variable. Although the underlying hard tissue can be reshaped by surgery (e.g. alveolar ridge augmentation and genioplasty), the exact position of the corresponding hard tissue point of the MdP is difficult to alter, since abrupt steps between the margin zone of the alveolus and the mental protuberance should be avoided.

Justin *et al.*^29^ quantified the extent of movement of the lower face during smiling. Their analysis, however, was merely based on some discrete landmarks limited to the mouth and alar base without curve fitting analysis. This study included points from the cheekbone to below the mouth. Our results showed the range of movement of the right contour is larger than the left when smiling and laughing, indicating more animation on the right side. The right contour line has more prominent differences between the stable area and the moving area of this smile-related curve contour during smiling.

On the basis of the canonical correlation analysis, there was a strong correlation between the soft tissue and hard tissue positions of L1′/R1′ (MxP), L3′/R3, and L2′ (CP), all of which were located on the stable zone of the cheekbone contour. The results indicate that the underlying hard tissue configuration of these areas determines the appearance of soft tissue in the static state as well as in the smiling and laughing states. Therefore, skeleton reshaping will change both the static and dynamic esthetics of these areas. The area around the mouth corner (L4′/R4′) and the middle zone of right cheekbone contour (R2′) both display close relationships to the smile/laugh movements. At L4′/R4′ and R2′, strong correlations were found with the position of the underlying hard tissue. The results suggest that the surface morphology of these areas is shaped mainly by the hard tissue configuration in the static condition but changes variably during smiling or laughing. Thus, altering esthetics of these areas requires hard tissue changes as well as muscle training.

## Additional Information

**How to cite this article**: Lin, Y. *et al.* A novel three-dimensional smile analysis based on dynamic evaluation of facial curve contour. *Sci. Rep.* **6**, 22103; doi: 10.1038/srep22103 (2016).

## Figures and Tables

**Table 1:** Definition of three assistant soft tissue landmarks.

Landmark	Abbreviation	Definition
Cheekbone point	CP	Point at the apex of osseous cheekbone located 20–25 mm inferior and 5–10 mm anterior to the outer canthus of the eye in sagittal view and is 20–25 mm inferior and 5–10 mm lateral to the outer canthus of the eye in frontal view.
Maxilla point	MxP	Most anterior point on the continuum of the cheekbone-nasal-lip contour and directly posterior to the alar base described by Arnett and Bergman^10^,^11^. It is an indicator of maxillary anteroposterior position.
Mandible point	MdP	Point located at the junction among the lateral subunit of cheek unit, central subunit of lower lip unit and mental unit, according to the classification of facial aesthetic units. It is normally directed at the end of mentolabial sulcus and nasolabial fold. Therefore it was so named by the author.

**Table 2:** Coefficient estimates of curve-fitting equation.

	Static		Smile		Laugh	
	L	R	L	R	L	R
*a*_1_	0.4127	0.0492	0.3210	0.3527	0.0648	0.0687
*a*_2_	1.5194	0.7027	2.0286	2.0065	2.0998	2.1440
*a*_3_	−0.0645	−0.3672	−0.0834	0.4302	0.1748	−0.1168
*a*_4_	−0.6491	−0.1078	−0.6452	−0.8036	−0.2387	−0.1322
*a*_5_	0.0046	−0.1639	0.1432	0.3103	0.0004	−0.1361
*a*_6_	−1.1318	0.8121	−1.8560	−1.7602	−1.4606	−1.5442
*a*_7_	−0.1936	−0.4476	−0.4187	−0.0624	−0.2149	0.1794

L: left side, R: right side.

**Table 3:** Motion amplitude and location of specific points on the Cheekbone-Maxilla-Nasolabial contour.

Error bar	Smile (left)		Smile (right)		Laugh (left)		Laugh (right)	
	Amplitude (mm)	Curvilinear distance (%)	Amplitude (mm)	Curvilinear distance (%)	Amplitude (mm)	Curvilinear distance (%)	Amplitude (mm)	Curvilinear distance (%)
1	4.5633 ± 2.7769	0	3.4502 ± 2.8497	0	5.6631 ± 2.3941	0	4.2619 ± 2.66074	0
2	4.2685 ± 3.1600	11.43 ± 3.02	7.4035 ± 7.0438	12.83 ± 6.01	7.9313 ± 2.7282	29.36 ± 2.99	10.4116 ± 4.5364	28.98 ± 2.50
3	3.9661 ± 1.7609	42.69 ± 3.81	5.0588 ± 2.3507	47.75 ± 4.38	6.7510 ± 2.8830	43.52 ± 3.14	6.7892 ± 2.9112	45.6 ± 2.61
4	8.9705 ± 3.8723	73.19 ± 3.88	9.9025 ± 2.7211	76.78 ± 2.63	11.8696 ± 3.2113	79.48 ± 1.96	12.2758 ± 2.8916	78.94 ± 1.81
5	4.5297 ± 2.2382	100	5.0462 ± 3.1000	100	5.4605 ± 2.3870	100	5.9938 ± 3.1283	100

**Table 4:** Canonical correlation analysis between specific soft tissue and hard tissue landmarks on the Cheekbone-Maxilla-Nasolabial contour.

Aspect	Number	Canonical correlations	Squared canonical correlations	Test of significance		
				Wilk’s L.	*F*	*P*
L1′	1	0.764	0.584	0.229	11.705	<0.001
	2	0.666	0.444	0.552	9.174	<0.001
L2′	1	0.853	0.728	0.147	16.878	<0.001
	2	0.677	0.458	0.541	9.527	<0.001
L3′	1	0.813	0.662	0.093	23.240	<0.001
	2	0.755	0.571	0.275	23.989	<0.001
L4′	1	0.799	0.638	0.282	9.605	<0.001
	2	0.380	0.145	0.778	3.541	0.009
L5′	1	0.332	0.110	0.862	0.886	0.539
	2	0.162	0.026	0.969	0.423	0.792
	3	0.070	0.005	0.995	0.268	0.607
R1′	1	0.652	0.425	0.552	3.898	<0.001
	2	0.202	0.041	0.959	0.562	0.691
R2′	1	0.895	0.800	0.057	31.467	<0.001
	2	0.750	0.563	0.288	22.900	<0.001
R3′	1	0.919	0.845	0.052	33.548	<0.001
	2	0.687	0.473	0.333	19.424	<0.001
R4′	1	0.820	0.672	0.207	12.791	<0.001
	2	0.526	0.276	0.633	6.813	<0.001
R5′	1	0.395	0.156	0.796	1.384	0.202
	2	0.203	0.041	0.943	0.783	0.538
	3	0.126	0.016	0.984	0.876	0.353
